# SAI-RRT*: Safety-Aware Informed RRT* for Multi-Joint Manipulator Path Planning in Static Environments

**DOI:** 10.3390/s26144490

**Published:** 2026-07-15

**Authors:** Zili Wang, Zhuo Wang, Xiaoru Li, Caichuang Wang

**Affiliations:** 1 School of Mechanical Engineering, University of Shanghai for Science and Technology, Shanghai 200093, China; wangzilict@163.com; 2 School of Mechanical Engineering, Nantong University, Nantong 226019, China; wangcaichuang@163.com

**Keywords:** SAI-RRT*, adaptive constrained circular area sampling, joint constraint framework, self-collision avoidance

## Abstract

**Highlights:**

**What are the main findings?**

- The proposed SAI-RRT* enhances Informed RRT* through a coordinated safety-aware planning loop that couples adaptive constrained sampling, DQN-based local refinement, and full-body geometric verification.
- Numerical simulations demonstrate that SAI-RRT* achieves stable and efficient path planning with improved geometric safety in static constrained environments.

**What are the implications of the main findings?**

- SAI-RRT* provides a planning-level method for improving geometric safety and planning efficiency in industrial multi-joint manipulator path planning.
- The algorithm’s core strategies provide a planning-level framework for improving sampling efficiency and geometric safety in tested static environments.

**Abstract:**

Path planning for multi-joint robotic manipulators within complex industrial settings represents a formidable challenge due to high-dimensional spaces and safety requirements. While the conventional Informed RRT* provides a solid foundation, it frequently encounters bottlenecks such as low initial sampling efficiency and poor adaptability. To overcome these issues, this study proposes SAI-RRT* (Safety-Aware Informed RRT*), a specialized framework designed to improve planning efficiency and geometric collision safety for 6-DOF multi-joint manipulator planning through an optimization strategy. First, we replace traditional global exploration with an adaptive constrained circular area sampling technique to reduce invalid exploration and accelerate initial path discovery. Second, a joint constraint framework combining kinematic verification and local reinforcement learning is applied to adjust joint configurations to avoid node rejection. Third, forward-kinematics-based full-body geometric verification is embedded into the tree expansion process, where the capsule model serves as an efficient link-clearance checking layer. Numerical experiments under static obstacle conditions show that SAI-RRT* improves planning efficiency, geometric collision safety, and path quality.

## 1. Introduction

In constrained industrial environments, including automated assembly lines, precision processing workshops and dense material handling areas, multi-joint manipulators usually work in cluttered workspaces filled with fixed machinery, tooling fixtures, conveyor belts, and even simultaneous operators. Such working scenarios require precise and collision-free trajectory planning within a high-dimensional configuration space, where the movements of each joint interact in a nonlinear manner, thus defining the overall pose of the arm. For robotic systems with 6 degrees of freedom (DOF), the dimension of configuration space increases exponentially, which brings great difficulties to the comprehensive balance of trajectory accuracy, operation safety and task execution efficiency. Conventional motion planning approaches, from sampling-based methods, heuristic methods, to intelligent optimization-based methods, mostly take end-effector path planning as the core objective, merely concentrating on guiding the end-effector to reach the target position while neglecting full-body obstacle avoidance and singular pose problems of multi-joint manipulators. This oversight may lead to collisions between intermediate links, joints, or the manipulator base and surrounding obstacles, as well as self-collisions, further triggering equipment failure, production suspension, or safety risks in human–robot collaboration scenarios. In addition, these traditional algorithms usually suffer from critical limitations. Due to the inefficiency of redundant sampling or search strategies in unpromising areas, the computation time is too long to meet the real-time requirements of industrial tasks [1,2,3]. In complex scenarios involving narrow passages or strict kinematic constraints, the above defects become more prominent, making traditional methods inadequate for the needs of modern intelligent manufacturing.

At present, path planning algorithms are mainly divided into three categories according to their basic principles and implementation logic: heuristic methods, sampling-based methods and intelligent optimization-based methods.

Heuristic methods represented by Dijkstra and A* employ structured search strategies to find the optimal path in discrete environments. The Dijkstra algorithm systematically explores the map from the starting point, ensuring the shortest path in the weighted space by prioritizing nodes with the lowest cumulative cost. A* is an extension of the Dijkstra algorithm, which combines heuristic functions to estimate the distance to the target and improve efficiency in low-dimensional static scenes by directing exploration. However, these methods struggle with the exponential complexity of high-dimensional space, which often leads to excessive computation time and limited adaptability to dense environments. Tang et al. [4] integrated an improved A* algorithm with the artificial potential field-based posture adjustment method, and proposed a global obstacle avoidance path planning approach for 6-DOF robotic arms. He et al. [5] presented an improved joint space A* algorithm, which adopts a pre-planning strategy for a 6-DOF robotic arm to solve the problem of high computational load and path instability in conventional link-wise obstacle avoidance. Even with these improvements, these algorithms still cannot meet the customized planning requirements of complex application scenarios. Moreover, both A* and Dijkstra exhibit evident deficiencies in high-dimensional adaptation, making them difficult to handle the multi-DOF configuration space inherent in path planning.

The second category comprises sampling-based methods, and classic examples include PRM [6,7], RRT [8] and EST [9]. Such methods do not require a complete modeling of high-dimensional spaces. Instead, they accomplish path planning via random node sampling and feasible path connection, thereby delivering superior high-dimensional capability and robust adaptability. Jamshidi et al. [10] proposed an enhanced version of the PRM algorithm for path planning in 3D space, which not only achieves the shortest path but also improves execution speed and the ease of implementation. Zhang et al. [11] proposed an improved PRM algorithm for dual-arm robot path planning, which adopts a normal-distribution-based sampling strategy integrated with adaptive perturbation and redundancy compression to enhance sampling quality, reduce redundant nodes, shorten paths, and maintain collision-free performance. Gong et al. [12] proposed the RL_PRM algorithm based on a restricted sampling area and lazy collision idea to address PRM’s shortcomings, which achieves faster convergence speed, shorter path lengths and fewer sampling points. Chen et al. [13] proposed the A-RRT* algorithm by integrating A* path-guided adaptive sampling, a goal bias strategy, and parent node reselection based on steering angles. Uzun et al. [14] proposed the n-Sliced Informed RRT* (nSIRRT*) to mitigate the poor performance of Informed RRT* in multi-curve path scenarios. Mashayekhi et al. [15] developed Informed RRT*-Connect by incorporating informed sampling into the RRT*-Connect framework. Jiang et al. [16] proposed the BUG0-IRRT* algorithm, which embeds the Bug0 algorithm for initial path generation and a grid-based node storage mechanism into Informed RRT*. Li et al. [17] presented an enhanced Informed RRT* algorithm integrated with the APF method to improve planning efficiency and path quality. Xiong et al. [18] proposed an improved Informed RRT* algorithm that integrates greedy strategies, adaptive step sizes, improved artificial potential field methods and optimization based on third-points. Guo et al. [19] proposed an efficient path planning algorithm based on adaptive sampling and dynamically constructed local informed sets, which boosts sampling efficiency and convergence speed for robotic path planning tasks. Fu et al. [20] proposed an improved RRT* algorithm equipped with a novel two-stage collision detection model that combines Mahalanobis distance and GJK algorithm, enabling efficient and accurate 3D collision checking for robotic arms. Wang et al. [21] proposed an improved Informed RRT* integrated with adaptive ellipsoidal sampling and DWA-based local planning, achieving smoother and more efficient trajectories in dynamic 3D underwater environments. Hong et al. [22] proposed VG-RRT*, which leverages a Voronoi-skeleton-guided sampling strategy to improve exploration efficiency in narrow environments and reduce redundant node generation. Kang et al. [23] proposed Smooth-RRT*, which introduces a reconnection-based smoothing strategy under kinodynamic constraints to generate smoother and more feasible trajectories while maintaining asymptotic optimality. Chen et al. [24] proposed an improved Informed RRT* with adaptive Gaussian mixture sampling and step-size adjustment to enhance path quality and planning efficiency in large-scale environments. However, despite continuous improvements in various aspects such as sampling strategies, exploration guidance, and trajectory smoothing, these methods generally remain heuristic in nature and may not guarantee global path optimality. Moreover, the inherent randomness of sampling-based search may lead to uneven node distribution, resulting in redundant node generation in certain regions of the configuration space. This issue becomes more pronounced in complex environments with highly non-uniform obstacle distributions, where planning performance is sensitive to environmental geometry.

The third category is a method based on intelligent optimization, which uses evolutionary computing, swarm intelligence or machine learning mechanisms to optimize the movement trajectory. Dorigo et al. [25] pioneered the ant system paradigm inspired by ant colony behavior, which combines positive feedback, distributed computation and constructive greedy heuristic algorithms to solve random combinatorial optimization problems. Kennedy et al. [26] put forward the Particle Swarm Optimization (PSO) and demonstrated its application in function optimization and neural network training. Kumaar et al. [27] designed a reinforcement learning-based path planner for dynamic environments, which uses deep Q-learning to acquire the initial path to adapt to time-varying environmental conditions. Tariq et al. [28] proposed a mobile robot path planning method based on reinforcement learning, which uses the Soft Actor-Critic Lagrangian (SACL-L) framework and combines the collision probability to optimize the reward function design. However, existing intelligent optimization-based methods still confront significant challenges in adapting to the high-dimensional configuration space and constraint-limited industrial scenarios of multi-joint manipulators. They find it difficult to perform local movement refinement while coordinating nonlinear coupling between multiple joints. Furthermore, they lack an effective mechanism to verify the precise pose of intermediate connecting links, fail to fully alleviate collision risks in complex movements, and are inadequate in meeting the strict kinematic constraints of multi-joint structures. Nevertheless, their innovative improvements have laid a solid foundation for further research on more robust and efficient path planning algorithms.

This paper adopts a sampling-based method to study the high-dimensional path planning problem of robotic arms. In the field of path planning, the Rapidly Exploring Random Tree (RRT) algorithm has become a well-recognized classic method after years of development. Continuous optimization efforts have been devoted to RRT variants, with the primary focus placed on enhancing planning efficiency and high-dimensional exploration capability. As one of the many improved versions of RRT, Informed RRT* [29] occupies an important position with its unique design. This algorithm innovatively constructs an elliptical sampling area, which is defined by the starting point, target point, and the currently discovered optimal path. This design significantly reduces invalid search behavior and accelerates convergence toward the global optimum. However, when the standard Informed RRT* algorithm is applied to high-dimensional joint space path planning of multi-joint manipulators with 6 DOF, its inherent shortcomings become prominent. The global random sampling mode used in the initial stage of the algorithm cannot rapidly generate a feasible initial trajectory, thereby resulting in low planning efficiency. Moreover, the algorithm lacks a rigorous verification mechanism for intermediate joint angles and link postures, which makes it prone to safety hazards such as self-collisions or collisions between the connecting links and surrounding obstacles during the movement of the manipulator.

Considering the state of the art and the limitations of existing algorithms in manipulator path planning, this paper aims to address two key issues: slow convergence speed during the initial sampling process and poor adaptability in high-dimensional space. Accordingly, this article proposes an enhanced path planning algorithm, which is called SAI-RRT* (Safety-Aware Informed RRT*). It is designed to extend the application of Informed RRT* to high-dimensional multi-joint planning scenarios to multi-joint systems through a coordinated safety-aware planning mechanism. First, adaptive constrained circular region sampling is adopted to replace traditional initial global random sampling, compress the invalid exploration scope to the maximum extent, and accelerate the generation of initial effective paths. Second, a joint constraint framework integrating kinematic verification and local reinforcement learning is constructed to dynamically adjust joint configuration parameters and remarkably lower the invalid judgment rate of sampling nodes. Third, a forward-kinematics-based full-body verification layer is embedded during tree expansion to evaluate intermediate-link clearance and self-collision risk. The combination of the above optimization strategies improves planning stability and efficiency in static, cluttered, high-dimensional manipulator planning scenarios.

## 2. Related Work

### 2.1. Evolution of RRT-Based Path Planning

#### 2.1.1. RRT

Since the pioneering RRT algorithm [8], the sampling-based motion planner has made great progress, which has laid a solid foundation for the path planning algorithm. In each iteration, the algorithm randomly samples a state from the entire state space, uses a distance metric to identify the nearest existing vertex in the current tree, and selects a control input to drive the nearest vertex to move slightly to the state of random sampling. Once a new state is obtained via such control input, it will be added to the tree and connected to the nearest vertex on the premise that it is located in the collision-free region.

However, RRT still has inherent limitations: its completely random sampling strategy leads to excessive ineffective exploration in a complex environment, which causes excessive time consumption during path discovery. Moreover, its handling of joint constraints and collision detection is still relatively general, and it fails to systematically solve the specific requirements of multi-joint robotic arms.

#### 2.1.2. RRT*

RRT* [30] is a landmark optimized variant of RRT. It retains the core incremental tree expansion mechanism while providing asymptotic optimality. Building on RRT’s sampling and nearest-neighbor search framework, RRT* introduces two key improvements: after generating a new state via steering the nearest vertex toward a random sample, it first identifies all nearby vertices within a dynamically adjusted connection radius and selects the parent vertex that minimizes the cumulative path cost to the new state. Second, it performs a “rewiring” operation to update the parent–child relationships of existing nearby vertices: if passing through the new state yields a lower-cost path to any nearby vertex, the tree structure is adjusted accordingly to maintain optimality.

Nevertheless, RRT* still has notable drawbacks in the context of multi-joint manipulator path planning. Its sampling strategy remains essentially random, leading to inefficient exploration in high-dimensional configuration spaces and failure to reduce invalid samples targeting obstacle regions. Although it improves path optimality, it does not fundamentally address joint constraints systematically and still uses relatively simplistic collision detection, failing to fully account for self-collision risks between intermediate links of multi-joint structures.

#### 2.1.3. Informed RRT*

**Figure 1:**
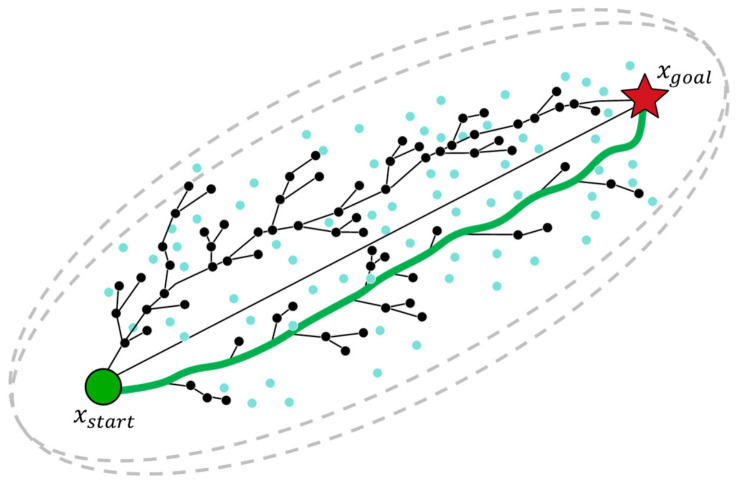
Informed RRT* Algorithm Schematic. The green circle in the figure represents the starting configuration ${\;x}_{start}$, while the red star denotes the target configuration $x_{goal}$, serving as the two foci of a slender ellipse (bounded by dashed lines). The cyan dots are sampling points of Informed RRT*, distributed solely within the ellipse. The black lines and nodes depict the RRT* tree structure, primarily expanding and reconnecting within the elliptical region. The bright green curve indicates an improved collision-free path with lower cost.

Informed RRT* [25] is a prominent optimization variant of RRT*. It retains the basic incremental tree extension and rewiring mechanism that supports RRT*, while significantly improving the speed of convergence to the optimal path. Building on the framework established by RRT*, Informed RRT* introduces a key informed sampling paradigm. After finding the initial feasible solution, it abandons uniform sampling of the entire state space. Instead, it constructs an elliptical subset of information focusing on the starting state and the target state, and the subsequent sampling process is carried out in the ellipse, as illustrated in Figure 1. Through this sampling method, the algorithm effectively eliminates the ineffective exploration of areas unrelated to solution optimization. Furthermore, as iterations increase, the solution cost gradually decreases, and the ellipsoid subset shrinks adaptively, thus forming a positive feedback loop and accelerating the convergence speed to the optimal path.

However, when applied to multi-joint manipulator path planning, the initial sampling of Informed RRT* is still carried out randomly in a global range, retaining a certain degree of randomness and inherent limitations. In addition, joint motion limits are still not systematically integrated into the sampling and state validation procedures. In addition, its collision detection mechanism remains limited to environmental obstacles, without fully accounting for the self-collision risk between intermediate links of multi-joint structures.

## 3. Methodology

Based on the limitations pointed out in the previous work, this section presents the SAI-RRT* algorithm, which adapts the standard Informed RRT* framework to safety-aware multi-joint robotic arm planning. By incorporating adaptive constrained circular-region sampling, DQN-based local joint refinement, and capsule-based full-body geometric verification, it significantly improves the initial path discovery efficiency, configuration feasibility, and self-collision safety, while preserving the asymptotic optimality of Informed RRT*.

### 3.1. Problem Formulation

The path planning problem of the multi-joint manipulator is defined as finding the optimal collision-free trajectory in the high-dimensional configuration space$\;C\subseteq R^{n}$ (where n≥6 DOF), from an initial configuration$\;q_{start}\in C_{free}\;$to a goal configuration $q_{goal}\in C_{free}.$

The configuration space C is divided into free space $C_{free}$ and obstacle space $C_{obs}$, where $C_{obs}$ includes environmental obstacles and self-collision regions. A feasible path is a continuous function $\tau :[0,1]\to C_{free}$ with $\tau (0)=q_{start}$ and $\tau (1)=q_{goal}$, satisfying kinematic constraints, singularity avoidance and precise end-effector pose requirements.

The objective of this paper is to minimize the path cost $c(\tau )=\int_{0}^{1}{∥\dot{\tau }(t)∥}_{2}\;dt\;$(Euclidean distance in joint space) under the premise of satisfying joint constraints and self-collision avoidance. Forward kinematics p=FK(q) maps configurations, the capsule model is used for collision detection, while inverse kinematics q=IK(p) assists in verification. High-dimensionality will aggravate the inefficiency of sampling and the risk of collision, and improvements in this paper aim to solve these problems. The symbols are shown in Table 1.

### 3.2. Adaptive Constrained Circular Region Sampling

In standard Informed RRT*, the initial global uniform sampling in high-dimensional space produces too many invalid samples, resulting in inefficiency. To accelerate the initial path discovery, adaptive constrained circular region sampling in the pre-information stage is introduced.

The proposed method does not perform uniform sampling over C, but limits the initial sampling to the circular area centered on the midpoint of the line segment connecting the initial point$\;x_{start}\;$and the goal point$\;x_{goal}$. This design leverages a simple geometric prior that feasible paths are more likely to lie near the straight-line connection between start and goal, especially in relatively open or moderately constrained environments. Uniform sampling in the unit sphere can be achieved by standard methods such as rejection sampling or spherical coordinate transformation, which can ensure the computational efficiency even in high-dimensional situations.

This constrained circular sampling is repeated until an initial feasible path connecting$\;x_{start}\;$to$\;x_{goal}\;$is found (i.e., a path with finite cost$\;c_{init}$). Once such a path is found, the algorithm switches to the standard Informed RRT* stage, in which the subsequent sample is extracted from the admissible ellipsoidal heuristic region defined by the current optimal path cost. The circular sampling region is formally defined as follows:

The midpoint of the start-goal line segment$\;x_{center}\;$is given by Equation (1):

(1) $$x_{center}=\frac{x_{start}+x_{goal}}{2}.$$

Equation (2) indicates that r is set to half of the Euclidean distance between the start and goal configurations:

(2) $$r=\frac{∥x_{goal}-x_{start}{∥}_{2}}{2}.$$

Equation (3) depicts that a random sample point$\;x_{rand}\;$in the initial phase is evenly extracted from the circular sphere:

(3) $$x_{rand}∼U\left(B(x_{center},r)\right),$$

where$\;B(x_{center},r)=\{x\in C|{∥x-x_{center}∥}_{2}\;\leq \;r\}\;$denotes the closed ball of radius r centered at$\;x_{center}$.

Equation (4) shows that in order to generate a uniform sample inside the sphere in a high-dimensional space, the algorithm first generates a point u uniformly distributed in the unit sphere B(0,1), and then scales it as follows:

(4) $$x_{rand}=x_{center}+r\cdot u,u∼U(B(0,1)).$$

An adaptive sampling strategy is designed to improve path-planning efficiency in complex environments. Its core idea is to dynamically adjust the sampling range according to real-time planning performance, rather than using a fixed sampling domain.

During the sampling phase, the algorithm initializes a relatively small constrained sampling ball centered between the start and goal configurations. The adaptive update rule is defined in Equation (5). When the planner fails consecutively to find valid nodes within this limited region, the algorithm automatically expands the sampling radius${\;r}_{k}\;$by a fixed scaling factor α. This adaptive expansion continues until$\;r_{k}\;$reaches a predefined maximum limit$\;r_{max}$. Once a valid sample is successfully generated, the failure counter is reset, and the sampler maintains a focused search within a compact region. Here,$\;f_{k}\;$denotes the number of consecutive sampling failures at iteration k, and $\;f_{th}\;$is the predefined threshold that triggers sampling radius expansion.

(5) $$r_{k}=\left\{\begin{array}{cc} \min\left(r_{k}\cdot \alpha ,r_{max}\right)\;\;\;\;\;\;\;\; & if\;f_{k}>f_{th}\\ r_{k} & otherwise \end{array}\right..$$

These values were selected based on preliminary trials in the sparse, narrow, and dense environments. The selection principle was to preserve the focused-search advantage of the constrained circular region during early exploration while still allowing the planner to recover broader exploration ability when repeated failures occur.

α controls how quickly the constrained sampling region is enlarged when repeated sampling failures occur. A larger α allows the planner to escape local infeasible regions more rapidly, but may weaken the guidance effect of constrained sampling by expanding the search region too aggressively. Conversely, a smaller α preserves a compact sampling region but may slow down recovery when the initial constrained region does not contain feasible configurations.

The maximum radius$\;r_{max}\;$is used to prevent unrestricted expansion of the constrained sampling region. It is determined according to the joint-space range of the manipulator and serves as an upper bound to ensure that the adaptive sampling strategy does not exceed the feasible configuration domain.

The failure threshold$\;f_{th}\;$determines how many consecutive unsuccessful sampling attempts are allowed before expanding the constrained region. A small$\;f_{th}\;$makes the planner expand the sampling region frequently, which improves escape capability but may reduce the efficiency of focused sampling. A large$\;f_{th}\;$maintains a narrow sampling region for longer, which improves local exploitation but may cause stagnation in highly constrained environments.

This mechanism ensures that the planner quickly narrows down the search space when feasible paths exist, while gradually relaxing constraints to escape local minima when necessary. Consequently, the convergence speed and planning stability are significantly improved compared with conventional fixed-range sampling methods.

**Figure 2:**
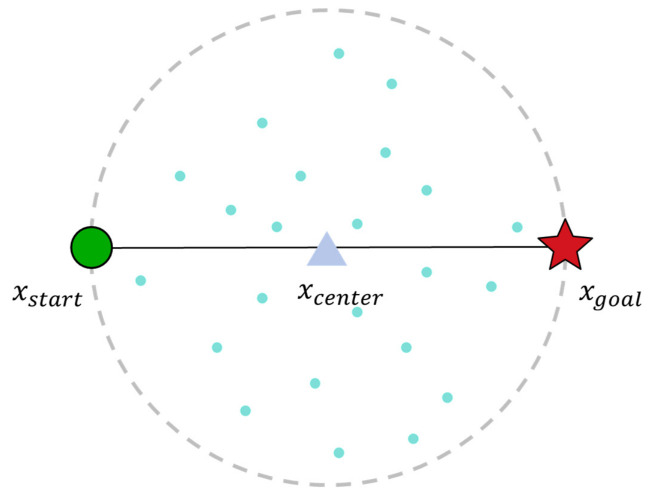
2D schematic diagram of the constrained circular sampling region in the initial phase of SAI-RRT*. The starting point $x_{start}$, goal point $x_{goal}$, and midpoint $x_{center}$ are marked, with the sampling ball shown in gray, with radius $r=\frac{∥x_{goal}-x_{start}{∥}_{2}}{2}$. The cyan dots are sampling points of SAI-RRT*.

Figure 2 shows the 2D schematic diagram of the constrained circular sampling region in the initial phase. This two-phase sampling strategy forms the basis for the subsequent joint constraint framework and pose verification, enabling efficient tree growth in high-DOF multi-joint manipulator scenarios.

### 3.3. Joint Constraint System

Multi-joint configurations often violate restrictions or singularities, resulting in a high rejection rate in the process of tree expansion. The joint constraint framework in this paper dynamically verifies and improves configurations, converting invalid nodes into feasible ones to improve the tree-growth efficiency in the coupled joint space.

The proposed DQN-based local refinement is selected over alternative correction methods, such as direct rejection, projection methods, local optimization-based corrections, and Jacobian-based adjustments for its superior adaptability to the highly coupled and singularity-prone configuration space of multi-joint manipulators. Direct rejection is simple and computationally inexpensive, but it discards all locally invalid configurations, including those that could be recovered through small joint adjustments. This reduces the utilization of sampled nodes and may increase repeated sampling and tree expansion costs in constrained regions. Projection methods, such as projection to joint limits, often enforce rigid constraint satisfaction but lack flexibility in balancing joint limits and singularity avoidance; they tend to produce large joint jumps or fail in near-singular regions, making them unsuitable for continuous tree expansion in sampling-based planners. Local optimization-based corrections require explicit objective construction and iterative solving, introducing extra computational cost, and they also struggle with local optima in high-dimensional non-convex spaces. Jacobian-based adjustments are effective for end-effector pose tracking but lack robustness when handling simultaneous joint limits, self-collision risks, and kinematic singularities, and divergence frequently occurs in highly constrained configurations. In contrast, the lightweight DQN network learns a local correction policy from offline data to make small, stable joint adjustments that simultaneously satisfy joint limits, avoid singularities, and preserve the original tree-growing direction. It avoids explicit complex modeling and heavy online computation, making it well matched to the incremental, probabilistic sampling framework of SAI-RRT*. This design maintains low computational latency while significantly reducing node rejection rates, which is validated in subsequent simulation results.

In the proposed planning loop, the DQN module therefore serves as a lightweight local repair stage, performing bounded small-step corrections near the sampled configuration rather than replacing continuous trajectory optimization. Each refined configuration is accepted only after passing joint-limit verification, singularity checking, and capsule-based pose verification, ensuring that the discrete DQN actions are used only for local feasibility recovery within the planning framework.

**Figure 3:**
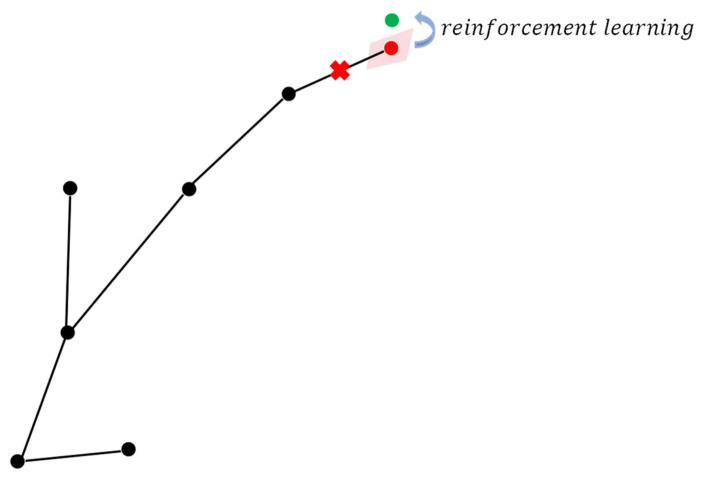
Schematic illustration of the joint constraint refinement process in SAI-RRT*. The red point denotes the initially invalid configuration, black points indicate the expanded nodes in the tree, blue arrows represent the reinforcement learning-based adjustment operations, the green point stands for the refined feasible configuration, and the red shaded areas indicate invalid regions, including physical limits and kinematic singularities.

The framework consists of two sequential stages: kinematic verification, and then local reinforcement learning improvement when necessary, as shown in Figure 3 of the overall algorithm process.

First, the kinematic verification of the newly generated configuration$\;q_{new}\;$is carried out immediately after the steering operation is completed. Two main constraints are checked at this stage:

For each joint i, q must satisfy

(6) $$q_{min,i}\leq q_{i}\leq q_{max,i}.$$

In addition, the Jacobian matrix J(q) is computed, and its condition number is evaluated to detect near-singular poses. If

(7) $$cond(J(q))>\epsilon_{COND},$$

the configuration is marked invalid, where$\;\epsilon_{COND}>0\;$is a predefined threshold. This threshold is chosen to prevent pathological inverse kinematics and numerical instability in the high-DOF manipulators.

Equation (6) shows that the joint-limit constraints ensure that the generated configuration stays within the physical range of each manipulator joint to prevent hardware violations or unreachable states during execution. The check is integrated early in the expansion stage, which can immediately reject obviously invalid samples and reduce computational waste in high-dimensional space.

Equation (7) describes the singularity avoidance criterion, where a large condition number signals configurations prone to instability, for example, small changes in joints leading to large end-effector movements. This threshold-based examination is crucial for multi-joint systems and is applied in parallel with joint limit verification. If it fails, it will trigger the improvement stage, which is able to maximize the utilization of nodes.

If the constraint in Equation (7) is violated, the framework applies local reinforcement learning (RL) for refinement instead of direct rejection. This paper uses a lightweight Deep Q-Network (DQN) agent to perform fine-grained adjustments to q. The reward function for the DQN agent is defined as follows, as illustrated in Equation (8):

(8) $$reward=-w_{1}\cdot d_{limit}-w_{2}\cdot s_{sing}-w_{3}\cdot c_{\tau }.$$

Equation (8) defines the reward function used to guide the DQN agent. First, $d_{limit}=\sum_{i=1}^{6}\left[max(q_{i}-q_{max,i},0)+max(q_{min,i}-q_{i},0)\right]$, which quantifies the total joint-limit violation distance and strongly penalizes configurations exceeding the physical joint limits of the manipulator. Second, $s_{sing}=max\left(0,\frac{\mathrm{cond}(J)-10}{50}\right)\;$is the normalized singularity penalty based on the condition number of the positional Jacobian matrix, penalizing configurations near kinematic singularities that may cause numerical instability and loss of dexterity. Third,$\;c_{\tau }\;$represents the estimated path cost from the current configuration to the goal. Overall, the reward function penalizes joint limit violations, near-singular configurations, and excessive path cost, while providing positive incentives for configurations that reduce the distance to the target. The weights $\;w_{1},w_{2},w_{3}>0\;$are set empirically, giving priority to feasibility over small cost increases. The equation encourages the agent to explore small adjustments Δq to restore effectiveness without deviating too far from the original guidance direction.

The action space of the proposed DQN refinement module is strictly discrete. For each of the six joints, three discrete actions are defined: decreasing the joint angle by a fixed small step, keeping the joint angle unchanged, or increasing the joint angle by the same step. Accordingly, the network outputs 18 Q-values corresponding to six joints with three candidate actions per joint. For each joint, the action associated with the maximum Q-value is selected independently. The selected actions are then applied simultaneously to all joints to produce a small local configuration adjustment.

The training of the DQN agent is carried out offline, using simulated configuration datasets from typical robotic arm tasks. The joint constraint refinement depicted in Algorithm 1 shows the complete process.
**Algorithm 1** Joint Constraint Refinement**Require:** $x_{rand}$, Maximum number of adjustment attempts $n_{max}$**Ensure:** Feasible joint configuration q* or reject for re-sampling  1: *Stage 1: Inverse Kinematics (IK) Solving*  2: $q_{candidate}\leftarrow \;$IK_Solve($x_{rand}$)  3: **if** IK_Success($q_{candidate}$) **then**
  4:   $q\;\leftarrow \;q_{candidate}$
  5: **else**
  6:   q ← INIT_JOINT_CONFIG()
  7: **end if**
  8: *Stage 2: RL-based Adjustment & FK Constraint Verification*
  9: **for**
*i* = 1 **to** $n_{max}$ **do**
 10:   **if not** IK_Success(q) **then**
 11:     δ ← RL_Agent_Adjust(q)
 12:     q ← max(min(q+δ, $q_{max}$), $q_{min}$)
 13:   **end if**
 14:   *Forward Kinematics (FK) Constraint Check*
 15:   $x_{curr}\leftarrow \;$FK_Compute(q)
 16:   **if** FK_Verify_Constraints ($x_{curr}$, $x_{rand}$) **then**
 17:     **return**
q
 18:   **end if**
 19: **end for**
 20: **return** reject

The local refinement module is referred to as DQN-based because it adopts a discrete action-value output structure for joint-level correction. However, in this implementation, it is used as a lightweight local refinement policy rather than a full temporal-difference DQN agent. Its purpose is not to learn a long-horizon sequential decision policy, but to provide fast and bounded joint corrections during RRT* tree expansion.

The input of the network is a 6-dimensional joint configuration, and the output layer contains 18 action scores, corresponding to three candidate actions for each of the six joints: decreasing the joint angle, keeping it unchanged, or increasing it by a fixed step. The network consists of two fully connected hidden layers with 128 neurons each, activated by ReLU functions. During online refinement, the action with the highest score is selected independently for each joint, and the selected six joint-level actions are applied simultaneously to produce a locally adjusted configuration.

The training samples are generated procedurally rather than collected from a fixed dataset. In each training epoch, one joint configuration is uniformly sampled within the joint limits of the manipulator, and one target configuration is also uniformly sampled to compute the path-cost term. Therefore, the training process uses 500 randomly generated joint configurations over 500 epochs. This uniform sampling strategy provides exploration over the joint-limit-bounded configuration space and exposes the network to different joint-limit and singularity-related conditions. Thus, the exploration strategy during offline training is uniform random exploration over the feasible joint range. The model is trained offline using the Adam optimizer with a learning rate of 0.0005.

The reward function penalizes joint-limit violation, singularity proximity, and path-cost increase, thereby encouraging small corrective actions that improve local feasibility while preserving the original tree-growing direction. Since the implemented module is a lightweight refinement policy rather than a standard temporal-difference DQN agent, no replay buffer, target network, discount factor, or mini-batch temporal-difference update is used. Equivalently, the update is performed on one procedurally generated sample per epoch, corresponding to an effective batch size of 1. Instead of optimizing a temporal-difference error, the network is trained by minimizing the negative differentiable surrogate reward, which is equivalent to maximizing the surrogate reward directly. This design keeps the refinement module computationally lightweight and suitable for repeated invocation during tree expansion.

During online planning, the trained network performs only feedforward inference. Greedy action selection is adopted by choosing the maximum-scoring action for each joint. Each locally invalid configuration is refined for at most three attempts. A refined configuration is accepted only after passing joint-limit verification, singularity checking, and capsule-based collision verification. No separate convergence threshold is used; training is terminated after the fixed 500 epochs. Because the training is performed offline before planning and only once for the tested manipulator model, its runtime is excluded from the online planning-time comparison. During online planning, only the feedforward inference time is counted.

In simulations, this framework converts a sizable proportion of initially invalid nodes into valid ones, greatly reducing the rejection rate and accelerating tree expansion in regions with tight joint coupling and high singularity risk.

### 3.4. Self-Collision Avoidance

Standard collision detection focuses on joint constraints but neglects self-collisions among intermediate links. This paper uses forward kinematics to improve obstacle avoidance via Cartesian pose verification.

For ${\;q}_{new}\;$or edge generated in the process of tree expansion, the algorithm uses forward kinematics to calculate the Cartesian position of all connecting links, as shown in Equation (9):

(9) $$p_{i}=FK(q_{new},i),i=1,2,\ldots ,m,$$

where FK(q,i) returns the position and orientation of the i-th link (including the end-effector as the last link), and m is the total number of links.

Two primary verification checks are then performed:

First, to prevent self-collision between non-adjacent links, this paper uses a capsule-based geometric model, an enhanced version of the discrete point-distance constraint:

(10) $$d_{capsule}(L_{i},L_{j})=max(0,dist(S_{i},S_{j})-(r_{i}+r_{j})),$$

where$\;dist(S_{i},S_{j})\;$represents the minimum Euclidean distance between segments$\;S_{i}\;$and$\;S_{j}$. As implemented in the SAI-RRT* framework, this is calculated by solving the optimization problem:

(11) $$dist(S_{i},S_{j})=min{∥(P_{i}+su)-(P_{j}+tv)∥}_{2},\;\;\;\;\;s,t\in [0,1],$$

where$\;P_{i}\;$is the segment endpoint and u,v are direction vectors. The minimum distance between any pair of non-adjacent links must be above a predefined safety threshold, evaluated using an enhanced capsule-based geometric model:

(12) $$\underset{i\neq j,|i-j|>1}{min}d_{capsule}(L_{i},L_{j})>\epsilon_{safe},$$

where$\;\epsilon_{safe}>0\;$is the minimum clearance required to prevent self-collision. Each link is modeled as a capsule$\;L_{i}$, defined by its finite three-dimensional central axis segment$\;S_{i}\;$and a characteristic radius$\;r_{i}$.

Second, the computed end-effector pose must satisfy the desired precision requirement:

(13) $$∥p_{end}-p_{desired}∥\;<\;\epsilon_{allow},$$

where$\;p_{end}=FK(q_{new},m)\;$is the end-effector pose,$\;p_{desired}\;$is the target pose (if specified), and$\;\epsilon_{allow}\;$is the allowable error, which can be divided into position error$\;\epsilon_{pos}\;$and rotation error$\;\epsilon_{rot}$.

Equation (10) defines the minimum clearance between two capsule-covered links, denoted as$\;d_{capsule}(L_{i},L_{j})$, by extending the conventional discrete point-distance constraint. Specifically, it calculates the shortest distance between the spatial segments$\;S_{i}\;$and$\;S_{j}\;$representing the central axes of the links, and then subtracts the sum of their respective characteristic radii,$\;r_{i}\;$and$\;r_{j}$. To ensure mathematical consistency, any negative values are capped at zero, which effectively indicates a physical penetration or intersection between the two capsule volumes. Equation (11) formulates the core geometric optimization problem used to determine the minimum Euclidean distance between the central axis segments. Each segment is mathematically parameterized using$\;P_{i}$, a normalized direction vector and a scalar parameter bounded within the interval [0, 1]. By minimizing the$\;L_{2}\;$norm of the difference vector between these two parameterized lines, the algorithm accurately captures the closest proximity between the two segments in the 3D Cartesian space, which serves as the foundation for the subsequent evaluation. Equation (12) establishes the final safety criterion for self-collision avoidance across the entire robotic structure. It enforces a strict constraint requiring that the minimum capsule distance between any pair of non-adjacent links must strictly exceed a predefined positive safety threshold$\;\epsilon_{safe}$. By ensuring that even the worst-case clearance among all eligible link pairs satisfies this inequality, the algorithm accepts a configuration only when the capsule-based clearance satisfies the predefined safety threshold during the tree expansion process. Equation (13) ensures the pose accuracy of the end-effector, and guarantees that the refined configuration can maintain the accuracy required for tasks such as grasping or assembly. This verification is particularly critical in high-DOF systems, where small joint changes will lead to noticeable end-effector drift.

**Figure 4:**
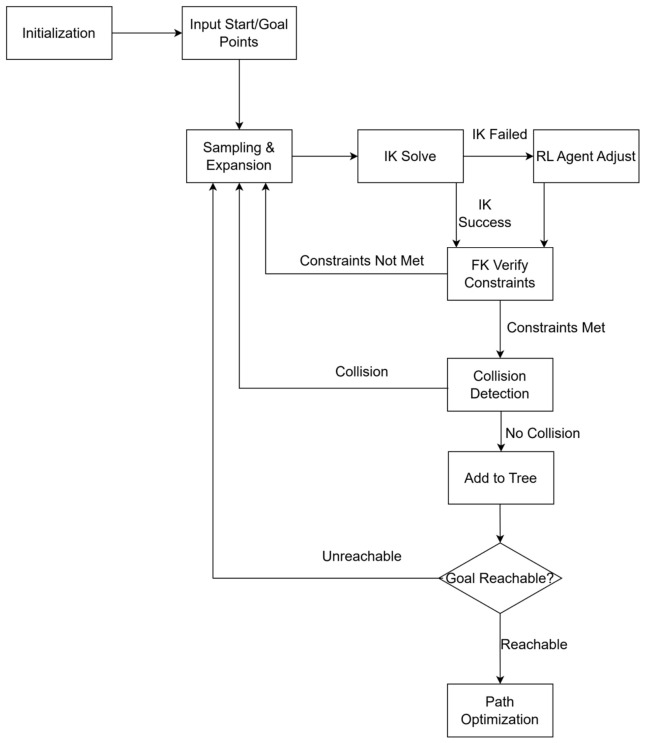
The algorithm flowchart.

In summary, combined with the three points put forward in this paper, the performance of Informed RRT* algorithm can be further improved in high-dimensional situations, for instance, enhancing the sampling efficiency and minimizing the corresponding collision risk. The algorithm flowchart is shown in Figure 4.

## 4. Simulation and Results

In this section, the proposed SAI-RRT* is compared with four existing algorithms: PRM*, MSSC-RRT*, standard Informed RRT* and RRT*. The experimental results show that the introduction of adaptive constrained circular region sampling in the initial sampling stage, the adaptation of Informed RRT* to safety-aware multi-joint manipulator planning, the improvement of the joint constraint framework and the use of full-body geometric verification for intermediate-link clearance checking improve the path quality and efficiency in static cluttered environments. All five algorithms are based on the parameter settings of Table 2.

For the simulation experiments conducted in this study, the PUMA 560 manipulator is adopted, whose standard Denavit-Hartenberg (DH) parameters are presented in Table 3, and the initial joint configuration and goal joint configuration adopt the standard zero pose and ready pose, respectively.

The experiments are conducted in a simulated 6-DOF manipulator environment, implemented on Windows using Python 3.13.5. The core path planning logic is developed entirely in Python, with the PUMA 560 robot model instantiated via the Robotics Toolbox for Python. Basic kinematics such as forward/inverse kinematics, Jacobian matrix computation, self-collision detection, and pose verification are all handled natively in Python using the roboticstoolbox and spatialmath libraries, while PyTorch 2.11.0 with CUDA 12.6 support is employed to implement the lightweight DQN agent for joint configuration refinement. All simulations are executed on an Intel Core i5-13500HX CPU with 16 GB RAM.

The design of these static environments is motivated by restricted-space industrial manipulation tasks, such as aerospace skin-panel wiring harness installation. In such applications, the manipulator operates around fixed structural elements, including brackets, stringers, and pre-routed harnesses, where the obstacle layout remains static during the installation process. The main planning challenge is to guide the manipulator through narrow gaps while maintaining sufficient clearance from surrounding structures and avoiding intermediate-link collision. Due to confidentiality requirements related to the specific industrial application, spherical obstacles are adopted as simplified geometric representations in the public simulation experiments. Therefore, the simulated spherical obstacles are used as simplified and repeatable geometric primitives to abstract restricted-space and narrow-passage conditions, rather than to fully reproduce all irregular industrial geometries.

The workspace is defined as a 3D Cartesian space with limits of [−1, 1] m (X-axis), [−1, 1] m (Y-axis), and [0, 1] m (Z-axis). Three experimental environments with increasing complexity are designed to evaluate the performance of SAI-RRT* against baseline algorithms:

- Sparse obstacles, with minimal clutter and clear navigable space;
- Narrow passages with moderate joint coupling;
- An environment characterized by densely distributed obstacles.

In Table 4, the algorithm evaluation is performed in accordance with the following evaluation protocol.

### 4.1. Environment A

**Figure 5:**
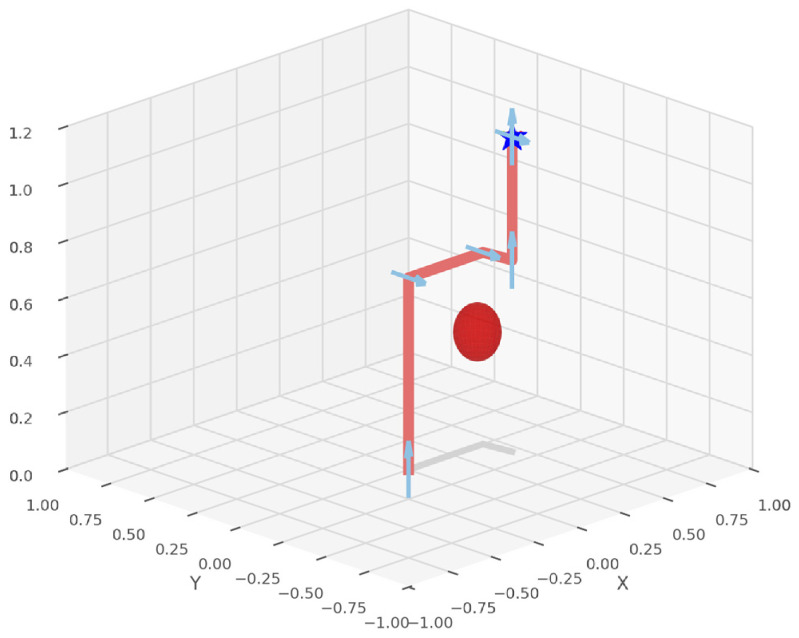
The first simulation environment at the start configuration. The blue arrows attached to the manipulator links denote the local coordinate frames of each joint. The red manipulator represents the initial joint configuration, serving as the starting point for the collision-free path generation, and the red sphere denotes the static obstacle, while the blue star marks the initial pose of the manipulator.

**Figure 6:**
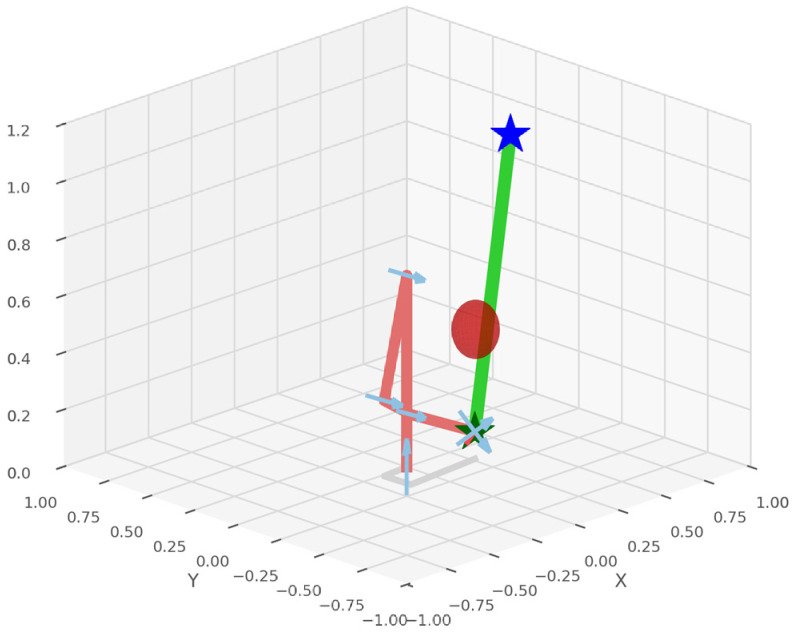
Result trajectory schematic diagram generated by SAI-RRT* based on environment A. The light green line represents the direct line segment connecting the initial and goal poses of the manipulator’s end-effector, while the dark green star indicates the target end-effector pose.

The first set of simulations is implemented in a sparse obstacle environment, which is composed of a single spherical obstacle located in the operational workspace of the 6-DOF robotic arm, as depicted in Figure 5. This experimental setup aims to simulate clutter-free operational scenarios with the least obstacles, mainly challenging the basic path-planning capabilities of the algorithm and validating its fundamental obstacle avoidance performance. Furthermore, the schematic diagram of the generated motion trajectory is shown in Figure 6, clearly demonstrating that the proposed SAI-RRT* algorithm can efficiently navigate around the isolated obstacle and reach the target pose accurately.

**Figure 7:**
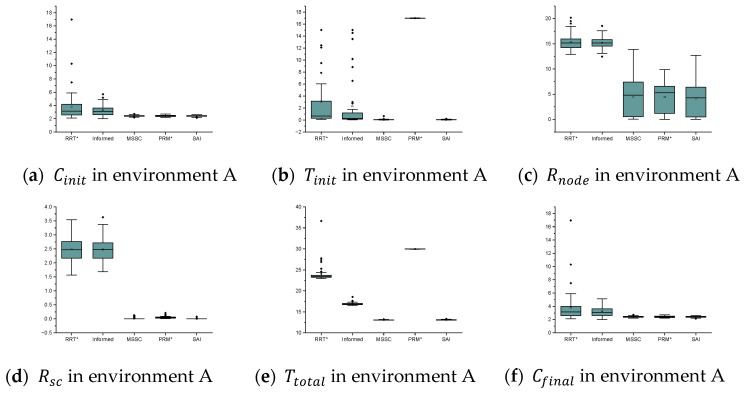
Performance indicators for the five algorithms within Environment A.

Figure 7 displays box plots summarizing the key performance indicators of the five algorithms in environment A. These figures include the cost of the initial feasible path$\;C_{init}$, time to find the first feasible path$\;T_{init}$, percentage of sampled nodes rejected due to joint constraints or singularities$\;R_{node}$, percentage of sampled nodes rejected due to self-collision detection$\;R_{sc}$, the total time spent on path planning$\;T_{total}$, and the final optimized path quality$\;C_{final}$.

Table 5 clearly documents the experimental results of RRT*, Informed RRT*, MSSC-RRT*, PRM*, and SAI-RRT* across independent simulation runs, evaluating their performance in terms of$\;C_{init}$,$\;T_{init}$,$\;R_{node}$,$\;R_{sc}$,$\;T_{total}\;$and$\;C_{final}$. Figure 7 evaluates the statistical performance of the five algorithms in the sparse environment and highlights the decisive advantages of the proposed SAI-RRT* over the other baselines. As shown in Figure 7a,b, SAI-RRT* achieves the lowest median and most compact distributions in both$\;C_{init}\;$and$\;T_{init}$, indicating its ability to generate high-quality initial feasible solutions with very low time consumption. Although MSSC-RRT* and PRM* also obtain relatively low$\;C_{init}\;$values, SAI-RRT* maintains a slightly better initial path cost while achieving the shortest initial solution time. In contrast, RRT*, Informed RRT*, and PRM* show larger$\;T_{init}\;$values, reflecting lower efficiency in quickly identifying feasible initial paths. Figure 7c,d show the rejection behavior caused by invalid nodes and self-collision risks. SAI-RRT* achieves the lowest$\;R_{node}\;$and$\;R_{sc}\;$among all algorithms, demonstrating that the proposed constrained sampling and capsule-based pose verification effectively reduce invalid node generation and suppress self-collision rejection. Compared with RRT* and Informed RRT*, which exhibit much higher rejection rates, SAI-RRT* provides a more stable and safety-aware tree expansion process. Based on Figure 7e, SAI-RRT* exhibits a decisive advantage over traditional baselines in$\;T_{total}$. SAI-RRT* and MSSC-RRT* deliver neck-and-neck performance with nearly identical low medians. Figure 7f illustrates the distribution of$\;C_{final}$. SAI-RRT* achieves this optimal performance with the most stable and compact distribution. It proves that SAI-RRT* provides stable, fast, and robust planning performance, effectively eliminating the high computational randomness inherent in traditional baseline planners.

From the data results, SAI-RRT* achieves the lowest$\;C_{init}\;$of 2.410 among all tested algorithms, slightly outperforming MSSC-RRT* with 2.411 and PRM* with 2.423, and showing a clear improvement over RRT* with 3.846 and Informed RRT* with 3.288. For initial solution efficiency, SAI-RRT* records the shortest$\;T_{init}\;$of 0.055 s, which is only 1.8% of RRT* and PRM* and 2.4% of Informed RRT*, while also being slightly lower than MSSC-RRT* with 0.057 s. This demonstrates that the proposed constrained sampling strategy can rapidly guide the search toward feasible and high-quality initial paths.

Regarding sampling validity, SAI-RRT* reduces$\;R_{node}\;$to 4.173%, which is significantly lower than RRT* with 15.358% and Informed RRT* with 15.219%, and also lower than MSSC-RRT* with 4.466% and PRM* with 4.498%. In terms of self-collision rejection, SAI-RRT* achieves the lowest$\;R_{sc}\;$of 0.003%, compared with RRT* with 2.483%, Informed RRT* with 2.481%, MSSC-RRT* with 0.010%, and PRM* with 0.049%. These results validate the effectiveness of the capsule-based pose verification in suppressing self-collision risks during tree expansion.

For total planning time, SAI-RRT* achieves a low$\;T_{total}\;$of 13.075 s, which is close to MSSC-RRT* with 13.066 s and substantially lower than RRT* with 24.020 s, Informed RRT* with 16.901 s, and PRM* with 29.975 s. Finally, SAI-RRT* obtains the lowest$\;C_{final}\;$of 2.404, outperforming RRT* with 3.812, Informed RRT* with 3.262, MSSC-RRT* with 2.408, and PRM* with 2.420. Overall, the results confirm that SAI-RRT* achieves superior initial path generation, lower invalid-node and self-collision rejection rates, and better final path quality while maintaining competitive computational efficiency in the sparse environment.

### 4.2. Environment B

**Figure 8:**
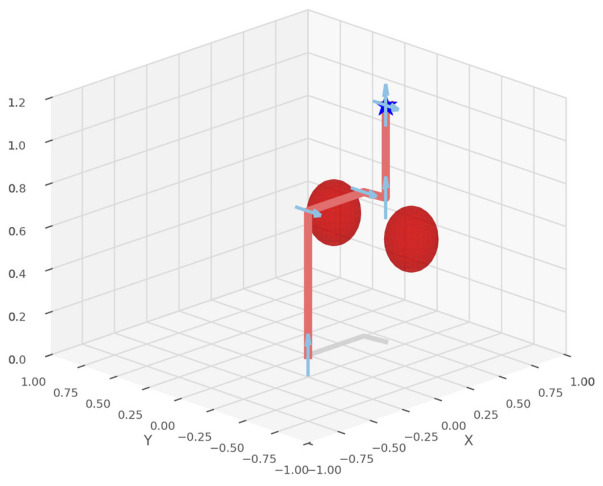
The second simulation environment at the start configuration.

**Figure 9:**
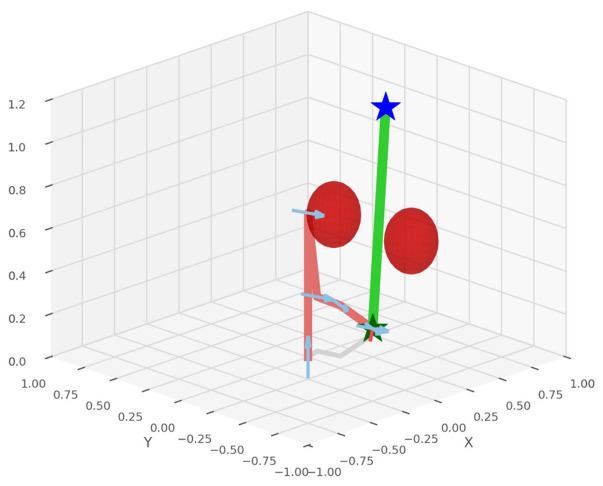
Result trajectory schematic diagram generated by SAI-RRT* based on environment B.

The second simulation is conducted in a narrow passage environment formed by two symmetrically placed spherical obstacles, creating a constrained corridor in the robotic arm’s workspace, which is shown in Figure 8. This configuration aims to simulate the restricted operating space that requires precise local path adjustment, thus putting forward strict requirements for the local optimization and constrained processing capacity of the algorithm. Additionally, the trajectory visualization schematic diagram displayed in Figure 9 shows that the algorithm can successfully identify the safe passage between the two obstacles, achieving collision-free navigation through the narrow corridor without violating joint limits or singularity constraints.

**Figure 10:**
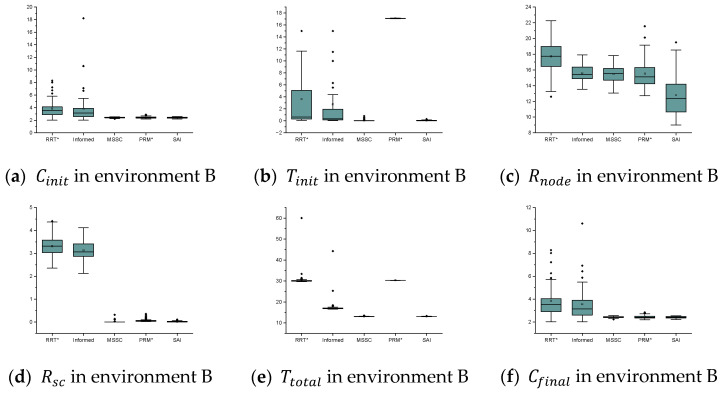
Performance indicators for the five algorithms within Environment B.

From the results shown in Figure 10 and Table 6, SAI-RRT* outperforms RRT* and Informed RRT* on all key metrics in Environment B. For$\;C_{init}$, SAI-RRT* achieves a lower cost of 2.417. As shown in Figure 10a, SAI-RRT* achieves the lowest distribution of$\;C_{init}\;$with a compact box range, indicating that it can consistently generate high-quality initial feasible paths under narrow-passage constraints. Figure 10b further shows that SAI-RRT* has the smallest$\;T_{init}\;$distribution, demonstrating its strong ability to rapidly obtain an initial solution despite the restricted search space. In Figure 10c, SAI-RRT* exhibits a lower$\;R_{node}\;$distribution than the baseline algorithms, which indicates that the proposed constrained sampling and DQN-based local refinement effectively reduce invalid node generation. The box plots in Figure 10d also maintain the lowest$\;R_{sc}\;$distribution, validating the effectiveness of capsule-based pose verification in suppressing self-collision risks. As shown in Figure 10e, SAI-RRT* achieves a low and stable$\;T_{total}\;$distribution, remaining close to MSSC-RRT* while clearly outperforming RRT*, Informed RRT*, and PRM*. Figure 10f illustrates that SAI-RRT* obtains the lowest$\;C_{final}\;$distribution, confirming that the proposed method maintains superior final path quality in the narrow environment.

SAI-RRT* achieves the lowest$\;C_{init}\;$of 2.417 among all tested algorithms, slightly outperforming MSSC-RRT* with 2.424 and PRM* with 2.442, and showing a clear improvement over RRT* with 3.869 and Informed RRT* with 3.815. For initial solution efficiency, SAI-RRT* records the shortest$\;T_{init}\;$of 0.068 s, which is only 1.9% of RRT*, 2.5% of Informed RRT*, and 0.4% of PRM*, while also slightly outperforming MSSC-RRT* with 0.072 s. This indicates that the proposed sampling strategy maintains strong initial path exploration capability even in the narrow environment.

Regarding invalid-node rejection, SAI-RRT* reduces$\;R_{node}\;$to 12.816%, which is lower than RRT* with 17.721%, Informed RRT* with 15.587%, MSSC-RRT* with 15.478%, and PRM* with 15.512%. For self-collision rejection, SAI-RRT* also achieves the lowest$\;R_{sc}\;$of 0.013%, compared with RRT* with 3.306%, Informed RRT* with 3.135%, MSSC-RRT* with 0.015%, and PRM* with 0.068%. These results demonstrate that the DQN-based local refinement and capsule-based pose verification effectively reduce invalid configurations and self-collision risks in constrained passages.

For total planning time, SAI-RRT* achieves a low$\;T_{total}\;$of 13.091 s, which is nearly identical to MSSC-RRT* with 13.072 s and much lower than RRT* with 30.781 s, Informed RRT* with 17.719 s, and PRM* with 30.312 s. Finally, SAI-RRT* obtains the lowest$\;C_{final}\;$of 2.412, outperforming RRT* with 3.844, Informed RRT* with 3.554, MSSC-RRT* with 2.421, and PRM* with 2.433. Overall, the results confirm that SAI-RRT* achieves the balance among initial path quality, search efficiency, invalid-node reduction, self-collision suppression, and final path in the narrow environment.

### 4.3. Environment C

**Figure 11:**
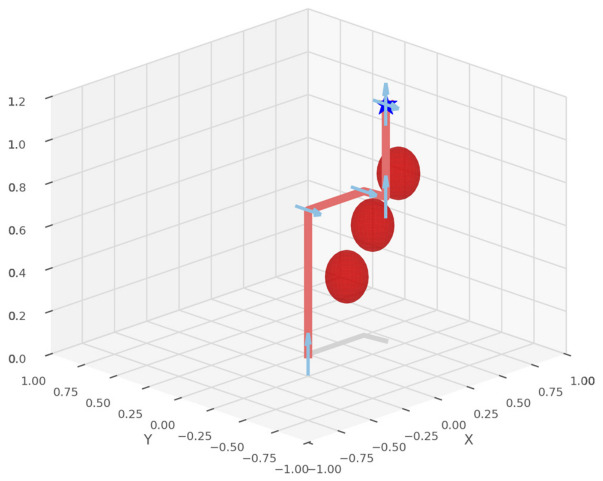
The third simulation environment at the start configuration.

**Figure 12:**
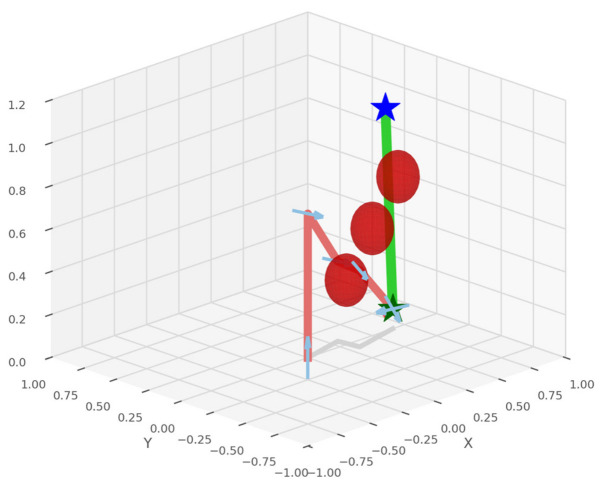
Result trajectory schematic diagram generated by SAI-RRT* based on environment C.

The third simulation is conducted in a dense obstacle environment composed of three sequentially arranged spherical obstacles distributed along the primary motion path of the robotic manipulator, as illustrated in Figure 11. This setting simulates a highly cluttered industrial environment with continuous obstacles, which poses serious challenges to the global path search efficiency and local obstacle avoidance accuracy of the planning algorithm. The schematic diagram of visualized planning path shown in Figure 12 confirms that the SAI-RRT* algorithm can effectively bypass all dense obstacles while maintaining path optimality and kinematic feasibility.

**Figure 13:**
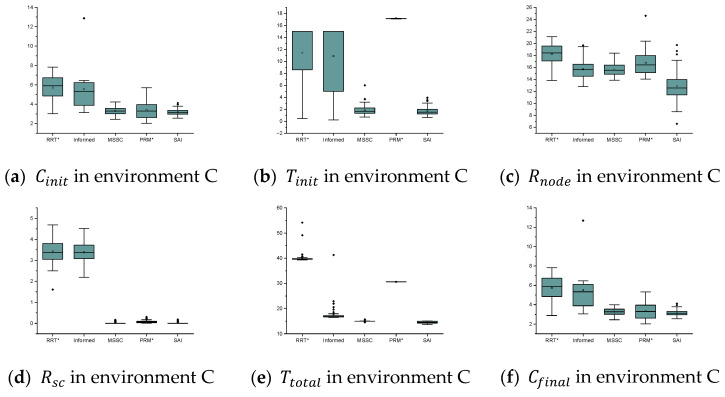
Performance indicators for the five algorithms within Environment C.

The relevant results under this environment are shown in Figure 13 and Table 7. For initial path quality$\;C_{init}$, SAI-RRT* achieves a mean value of 3.156, substantially lower than RRT* and Informed RRT*, and slightly outperforming MSSC-RRT*. Figure 13a shows that its box plot is tightly clustered at the bottom, confirming its ability to consistently generate high-quality initial solutions. In terms of computational efficiency$\;T_{init}$, SAI-RRT* records a mean of only 1.742 s, far shorter than the baselines. Compared with PRM*, which requires 17.132 s to obtain the initial feasible path, SAI-RRT* reduces$\;T_{init}\;$by nearly an order of magnitude, demonstrating a clear advantage in initial solution discovery. Figure 13b presents that the corresponding box plot exhibits a low median and compact spread, demonstrating fast and reliable initial path discovery. Regarding sampling quality, SAI-RRT* drops the mean$\;R_{node}\;$to 12.839, outperforming all other methods, as illustrated in Figure 13c. PRM* produces a higher$\;R_{node}\;$of 16.731, showing that its roadmap construction still generates more invalid configurations under dense obstacle constraints. Most significantly, for self-collision safety$\;R_{sc}$, SAI-RRT* maintains a negligible mean of 0.016, comparable to MSSC-RRT* but drastically lower than RRT* and Informed RRT*. As reflected in Figure 13d, its box plot is compressed flat near zero, validating the precision of its safety mechanisms. In addition, SAI-RRT* compresses the overall planning time$\;T_{total}\;$to 14.551 s, which is just 36.2% of RRT* and 81.4% of Informed RRT*. Compared with PRM*, whose$\;T_{total}\;$is 30.632 s, SAI-RRT* reduces the overall planning time to 47.5% of PRM*, showing significantly higher computational efficiency in the dense environment. Figure 13e indicates that the box plot of SAI-RRT* also shows a highly compact distribution. Finally, SAI-RRT* delivers the lowest final path cost$\;C_{final}\;$of 3.145 compared to MSSC-RRT*’s 3.299, RRT*’s 5.717, and Informed RRT*’s 5.522, as seen in Figure 13f. These characteristics highlight the superior stability, efficiency, and robustness of SAI-RRT*, even in the most challenging and cluttered workspace tested in this study.

### 4.4. Computational Cost and Trajectory Quality Analysis

#### 4.4.1. Computational Cost Analysis

This subsection analyzes the computational overhead introduced by the reinforcement learning joint adjustment module in the proposed SAI-RRT* algorithm. The RL cost is measured as the sum of the actual execution times of all calls to the RL function during each planning run. Averaged over 50 independent runs, the values are presented in Table 8. It can be seen that the RL overhead increases gradually with environment complexity, owing to the larger number of node adjustment attempts needed to avoid invalid configurations in more cluttered scenes. Despite this increase, the RL overhead remains very low compared with the total planning time. This small overhead is well justified by the significant reduction in the number of rejected nodes and the faster convergence to feasible and optimal paths.

#### 4.4.2. Runtime Breakdown

This subsection provides a detailed runtime breakdown of the proposed SAI-RRT* algorithm to further analyze its computational characteristics under the static planning conditions. As quantified in Table 9 and Table 10, the total planning time is decomposed into several major components, including sampling, nearest-neighbor search, collision checking and kinematic computation, DQN-based joint refinement, and path post-processing.

Table 9 presents the average runtime distribution of SAI-RRT* across different environments. In the sparse environment, the total planning time reaches 13.106 s, where sampling, nearest-neighbor search, collision checking and kinematic computation, DQN refinement, and path post-processing account for 0.035 s, 2.233 s, 1.654 s, 3.470 s, and 0.052 s, respectively. In the narrow environment, the total planning time slightly increases to 13.233 s, with corresponding component times of 0.033 s, 2.361 s, 2.096 s, 3.567 s, and 0.113 s. In the dense environment, the total planning time further increases to 14.298 s, while sampling, nearest-neighbor search, collision checking and kinematic computation, DQN refinement, and path post-processing account for 0.209 s, 2.908 s, 2.263 s, 4.287 s, and 0.167 s, respectively.

The results show that the main computational cost is concentrated in DQN-based refinement, nearest-neighbor search, and collision checking with kinematic computation. As the environment becomes more complex, the time consumed by nearest-neighbor search, collision checking and kinematic computation, and DQN refinement increases accordingly. In contrast, path post-processing remains relatively small in all three environments, indicating that the major computational burden occurs during online tree expansion rather than after path generation.

Table 10 further reports the runtime characteristics of the DQN-based joint refinement module. The DQN module is not invoked at every tree-expansion step, but only when a newly sampled or extended configuration violates joint constraints or approaches an invalid kinematic state. Across the sparse, narrow, and dense environments, the average numbers of DQN invocations are 1662.42, 1864.48, and 2013.72, respectively. The corresponding average execution times per invocation are 0.0021 s, 0.0019 s, and 0.0021 s, while the worst-case execution times are 0.0033 s, 0.0039 s, and 0.0044 s, respectively. These results indicate that although the DQN module is invoked more frequently in more complex environments, the runtime of each individual DQN call remains stable and bounded.

In terms of its functional contribution, the DQN fixed rates are 2.743%, 3.505%, and 3.780% in the sparse, narrow, and dense environments, respectively. The increasing fixed rate indicates that the DQN-based refinement contributes more noticeably when the planning environment becomes more constrained. Therefore, although the DQN-based refinement introduces a measurable runtime cost, it improves planning robustness by converting a portion of potentially invalid configurations into feasible ones instead of directly discarding them.

#### 4.4.3. Trajectory Quality Analysis

This subsection analyzes the safety margin and trajectory smoothness achieved by the proposed SAI-RRT* algorithm, as quantified in Table 11 and Table 12.

Table 11 presents the average minimum link clearance across different environments. The results show that SAI-RRT* consistently achieves the highest minimum link clearance in all scenarios. In the sparse environment, its clearance reaches 0.0081 m, which is significantly higher than that of RRT*, Informed RRT*, MSSC-RRT* and PRM*. Even in narrow and dense environments, SAI-RRT* maintains the largest clearance values, indicating that its improvements effectively push the manipulator links away from self-collision configurations, thereby improving safety robustness.

Table 12 reports the average trajectory smoothness. Compared with other algorithms, SAI-RRT* exhibits slightly higher values, indicating marginally greater joint angular variation along the path. This is likely a result of the safety-oriented refinement steps in SAI-RRT*, which prioritize configurations with higher minimum link clearance. While this introduces a minor trade-off in terms of smoothness, the resulting trajectory variation remains within a practically acceptable range. Nevertheless, this smoothness metric only evaluates geometric path continuity based on joint-angle variation, and should not be interpreted as a complete verification of dynamic feasibility. Additional time-parameterization and dynamic checks involving joint velocity, acceleration, jerk, torque, and payload constraints are required before direct industrial execution.

These results collectively highlight a clear trade-off between safety and smoothness. Although this safety-oriented design leads to slightly higher trajectory smoothness values, the resulting variation remains practically acceptable. SAI-RRT* balances geometric safety and path smoothness at the planning level, providing a useful basis for cluttered-environment manipulator planning before further dynamic feasibility validation.

### 4.5. Ablation Study

To isolate the contribution of each proposed component, an ablation study was conducted in the dense environment, which represents the most challenging scenario among the simulation environments, and each setting was evaluated over 50 independent runs. Four cumulative variants were evaluated: the original Informed RRT*, Informed RRT* with adaptive constrained circular sampling, Informed RRT* with adaptive constrained circular sampling and DQN refinement, and the full SAI-RRT* integrating adaptive sampling, DQN refinement, and capsule-based pose verification. The evaluation metrics include success rate, planning time, path length, invalid-node rate, clearance, smoothness, and collision-checking cost.

As shown in Table 13, the original Informed RRT* achieves a success rate of 80%, with a planning time of 18.425 s and a path length of 5.597. After introducing adaptive constrained circular sampling, the success rate increases to 84%, while the planning time is reduced to 11.386 s and the path length decreases to 3.391. This indicates that the adaptive constrained sampling strategy effectively improves exploration efficiency by guiding the sampling process toward more promising regions and reducing unnecessary global exploration.

After adding the DQN refinement module, the success rate further increases to 94%, and the invalid-node rate decreases from 17.427% to 13.473%. This demonstrates that the DQN-based local refinement contributes mainly to feasibility improvement by repairing part of the locally invalid configurations instead of directly discarding them. Although the planning time increases from 11.386 s to 14.109 s due to the additional refinement operations; the algorithm still remains faster than the original Informed RRT* baseline.

The full SAI-RRT* achieves the best overall performance, with the highest success rate of 96%, the shortest path length of 3.124, and the lowest invalid-node rate of 12.846%. Compared with the DQN-refined variant without capsule verification, the full SAI-RRT* further improves clearance from 0.0005 m to 0.0006 m, as shown in Table 14. This indicates that capsule-based pose verification enhances full-body collision safety by considering intermediate links and self-collision risks during tree expansion.

Table 14 further shows the trade-off between safety and computational cost. Adaptive constrained sampling reduces the collision-checking cost from 3.268 s to 1.946 s, indicating fewer ineffective collision-validation operations. After adding DQN refinement and capsule-based verification, the collision-checking cost increases to 2.148 s and 2.272 s, respectively, because additional local refinement and full-body pose verification are performed. However, these costs remain lower than that of the original Informed RRT*, while the success rate, path length, clearance, and invalid-node rate are improved. These results confirm that the final performance gain of SAI-RRT* is achieved through the coordinated contribution of adaptive sampling, DQN-based local refinement, and capsule-based collision verification.

## 5. Discussion

### 5.1. Analysis of Results

From the comprehensive results across three environments, the proposed SAI-RRT* exhibits consistent superiority over RRT*, Informed RRT*, and comparable or better performance than MSSC-RRT* and PRM* to some extents.

In all scenarios, the algorithm obtains lower$\;C_{init}\;$and shorter$\;T_{init}\;$with relatively compact box plot distributions, enabling fast, stable generation of high-quality initial solutions.$\;R_{node}\;$is effectively reduced and$\;R_{sc}\;$remains at an extremely low level, verifying the effectiveness of kinematic-aware sampling and capsule-based collision checking.

For$\;T_{total}$, SAI-RRT* achieves notable efficiency gains with concentrated distributions, demonstrating strong stability and robustness. Meanwhile, it consistently delivers the lowest$\;C_{final}$, achieving optimal path quality while balancing planning efficiency and solution optimality.

### 5.2. Advantages and Limitations

#### 5.2.1. Advantages

The advantages of SAI-RRT* are reflected in three key aspects. First, it is tailored for safety-aware planning of the 6-DOF multi-joint robotic arms. Its adaptive constrained sampling strategy compresses the sampling range via geometric priors, addressing core pain points like coupled joint-space exploration, invalid sampling, and intermediate-link collision risks. Second, it achieves collaborative multi-dimensional optimization. Adaptive constrained circular sampling accelerates convergence, the joint constraint framework enhances sampling effectiveness, and capsule-based verification improves planning-level geometric safety. At the same time, it addresses planning-level requirements for precise geometric path generation and collision safety, which traditional single-objective algorithms struggle to balance. Third, it shows improved adaptability across the static environments. Unlike RRT* and Informed RRT*, whose performance degrades significantly as complexity increases, it maintains stable and efficient operation.

#### 5.2.2. Limitations

Despite its significant advantages, SAI-RRT* still has certain limitations. First, it is currently applicable only to static obstacle environments. In real industrial scenarios, dynamic obstacles such as mobile machinery and human operators may occur, and the algorithm lacks real-time obstacle tracking and adaptive adjustment capabilities for changes, which limits its practical application scope. In addition, the current real-world validation is limited to static and known environments, where the trajectories are precomputed offline and executed in an open-loop manner. Therefore, robustness under perception noise, moving obstacles and online replanning has not yet been validated. Second, the reinforcement learning agent in the joint constraint framework uses offline training. The training dataset is derived from simulated configurations, which may deviate from the physical characteristics of real manipulators. This offline training mode makes it difficult for the agent to adapt to the uncertainties of real equipment, which may affect the optimization effect of invalid configurations in practical applications. Third, in terms of trajectory smoothness, the algorithm shows certain limitations compared with other methods, with relatively larger joint angular variations, which may impose higher requirements on the motion control system in practical deployment. Moreover, the current trajectory smoothness evaluation is based only on joint-angle variation and mainly reflects geometric path continuity. It does not fully verify dynamic feasibility under robot-specific velocity, acceleration, jerk, torque, or payload constraints. Therefore, although the proposed method improves planning-level collision safety and geometric feasibility, direct industrial execution still requires additional time-parameterization and dynamic feasibility checks. Future work will incorporate velocity and acceleration limits, jerk-continuous trajectory generation, torque constraints, and payload-dependent dynamic validation to further improve the deployability of the planned trajectories.

## 6. Real-World Experiments

### 6.1. Experiment Setup

To validate the practical applicability of the proposed motion planning algorithm in both simulated and physical environments, a comprehensive set of experiments was carried out using the AUBO-E5 6-DOF collaborative manipulator (AUBO Robotics, Beijing, China). This platform offers high positional repeatability, precise joint-level control, and a fully open kinematic architecture, making it ideal for preliminary evaluation of safety-aware multi-joint motion planning. The manipulator is controlled by a computer running Ubuntu 18.04, which serves as the middleware to provide a standardized framework for robot control, sensor data handling, and trajectory execution.

First, the experimental scenarios were established in simulation using ROS-based tools, with the AUBO-E5 kinematic model loaded alongside custom obstacle maps for three distinct configurations: sparse, narrow, and dense. The robot’s kinematic model strictly adheres to the manufacturer-provided DH parameters, ensuring a high degree of consistency between simulated and real-world behavior. It is worth noting that, as is standard practice, the DH-based model does not explicitly incorporate the physical dimensions of the end-effector gripper, which is instead treated as an external tool frame. This modeling convention maintains structural consistency while enabling efficient simulation-based planning.

For trajectory generation, collision-free paths were first obtained in simulation across the three environments using the proposed SAI-RRT* algorithm. Each node in the tree corresponds to a target joint configuration, and these six-dimensional joint angle sequences were sequentially sent to the manipulator controller for execution on the physical robot as a preliminary open-loop validation.

### 6.2. Experiment 1: Sparse Environment

**Figure 14:**
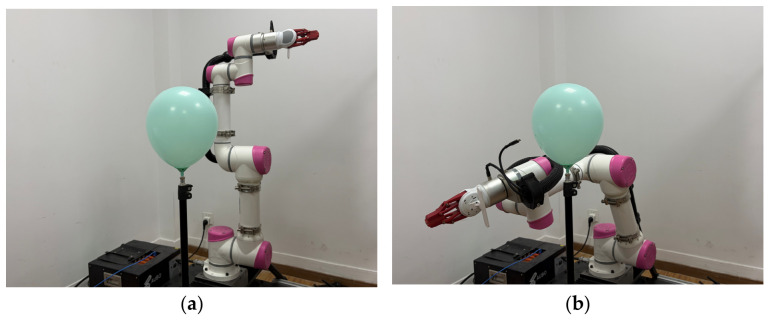
(**a**) The real-world sparse environment. (**b**) The planned trajectory result in the real-world sparse environment.

In the sparse environment, a single obstacle is placed with a height of 100 cm, leaving sufficient free space around it for the manipulator to navigate. The start configuration is set to the manipulator’s zero position pose, and the target position is at the opposite side of the obstacle. Figure 14a shows the real-world sparse environment, while Figure 14b depicts the path result. The planned trajectory, generated by the SAI-RRT* algorithm, was executed on the manipulator. The robot successfully avoided the obstacle without collision and reached the goal pose within the specified tolerance. The execution showed no significant deviation from the planned path, confirming the basic feasibility of the proposed algorithm in a low-obstacle scenario.

### 6.3. Experiment 2: Narrow Environment

**Figure 15:**
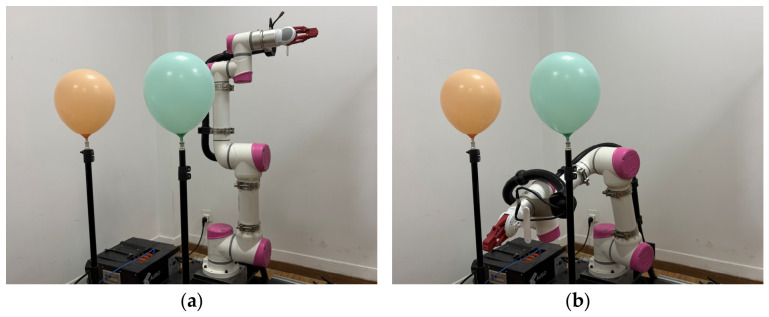
(**a**) The real-world narrow environment. (**b**) The planned trajectory result in the real-world narrow environment.

The narrow environment consists of two obstacles symmetrically placed along the robotic arm’s path, with a horizontal distance of 35 cm between them and a height of 110 cm above the ground. The passage formed by these two obstacles constitutes a strict constraint, requiring the robotic arm to traverse the narrow passage while maintaining a safe distance from both sides. The initial pose is the same as in the sparse environment, corresponding to the robotic arm’s zero-position pose, while the target pose is adjusted to require the end-effector to move to the center of the passage. In the actual environment, no collisions occurred during the execution of the planned path. The robotic arm adjusted its pose to pass through the narrow passage, demonstrating that the algorithm can handle strict spatial constraints. Due to model uncertainties, small tracking errors were observed, but these errors were within acceptable limits and did not affect task completion. The real-world narrow environment setup and the corresponding trajectory execution result are illustrated in Figure 15a and Figure 15b, respectively.

### 6.4. Experiment 3: Dense Environment

**Figure 16:**
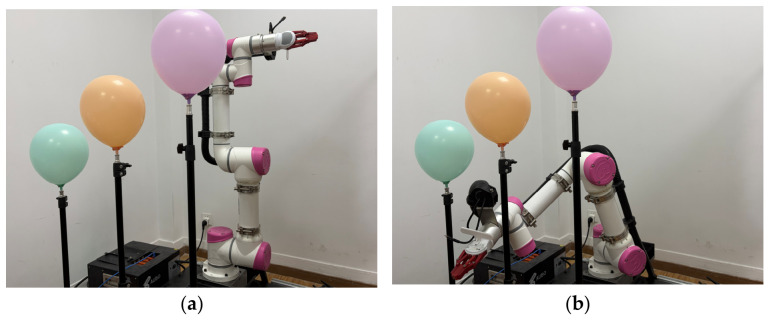
(**a**) The real-world dense environment. (**b**) The planned trajectory result in the real-world dense environment.

The dense environment features three obstacles distributed in sequence, forming a cluttered workspace with multiple potential collision points. Figure 16a shows the setup of the real-world dense environment, and Figure 16b presents the execution result of the planned trajectory. These obstacles are set with a gradient vertical height of 100 cm, 110 cm, and 120 cm, respectively, and the fixed spacing between every two adjacent obstacles is 25 cm, creating a compact and constrained operating space that poses high requirements for trajectory planning. The start configuration is the same as previous experiments, corresponding to the manipulator’s zero position pose, while the goal configuration requires the manipulator to pass through the passages formed between the adjacent obstacles, which demands a coordinated combination of horizontal and vertical movements. The proposed algorithm generated a collision-free trajectory that the manipulator followed successfully. The manipulator avoided all obstacles without any physical contact and completed the task. The execution provides preliminary evidence that the planned paths can be followed in a structured static high-obstacle-density scenario with gradient obstacle heights and narrow spacing constraints.

## 7. Conclusions

This study improves upon core issues of safety-aware multi-joint manipulator path planning for multi-joint manipulators in constrained industrial environments, where standard Informed RRT* suffers from low initial sampling efficiency, insufficient joint constraint handling, and incomplete self-collision avoidance. To overcome these limitations, this study proposes an enhanced algorithm, SAI-RRT*, which adapts the conventional Informed RRT* framework to 6-DOF multi-joint manipulator scenarios via three designed optimization strategies.

Practically, SAI-RRT* improves planning-level efficiency and geometric collision safety for industrial multi-joint manipulator path planning under static obstacle conditions. The results suggest its potential applicability to static industrial scenarios, such as automated assembly, precision machining, and high-density material handling, provided that subsequent time-parameterization and dynamic feasibility checks are performed. Future work will focus on extending the algorithm to dynamic obstacle environments, optimizing the reinforcement learning agent with online fine-tuning mechanisms, expanding its application to multi-robot collaborative planning scenarios and further improving trajectory smoothness. These extensions are expected to further improve the algorithm’s practicality and scalability, supporting its future application in increasingly complex intelligent manufacturing scenarios after dynamic feasibility validation.

## Figures and Tables

**Table 1:** Glossary of Key Symbols.

Symbol	Definition
C	Configuration space.
p	A task-space pose vector.
q	A joint configuration vector.
x	Notation for a point in the space.
r	Radius of constrained sampling circle.
τ	A continuous path from starting point to end point.
c(τ)	Path cost function.
FK(q)	Forward kinematics function.
IK(p)	Inverse kinematics function.
$\epsilon_{COND}$	condition number threshold.
$\epsilon_{allow}$	Pose error threshold.
$\epsilon_{safe}$	Safety threshold for self-collision avoidance.

**Table 2:** Parameter settings.

Parameter	Value
Step Size	0.5
Goal Bias	0.05
Maximum Initial Planning Time	20
Maximum Planning Time	60
Number of Runs	50
δ	0.01
$\epsilon_{safe}$	0.005
$\epsilon_{pos}$	0.12
$\epsilon_{rot}$	0.35
$\epsilon_{COND}$	30.0
$w_{1}$	1.0
$w_{2}$	1.0
$w_{3}$	2.2
RL Adjust Step	0.065
α	1.55
$f_{th}$	12

**Table 3:** DH Parameters of PUMA 560 manipulator.

Link i	*d_i_* (mm)	θ_*i*_ (rad)	*a_i_* (mm)	α_*i*_ (rad)
1	0	0	0	1.5708
2	0	0	431.8	0
3	150.05	0	20.32	−1.5708
4	431.8	0	0	1.5708
5	0	0	0	−1.5708
6	56	0	0	0

**Table 4:** Evaluation protocol.

Index	Definition
$C_{init}$	The cost of the initial feasible path.
$T_{init}$	Time to find the first feasible path.
$R_{node}$	Percentage of sampled nodes rejected due to joint constraints or singularities.
$R_{sc}$	Percentage of sampled nodes rejected due to self-collision detection.
$T_{total}$	The total time spent on path planning.
$C_{final}$	The final optimized path quality.

Relevant calculation formula: $R_{node}=\frac{N_{joint-rejection}}{N_{total}},R_{sc}=\frac{N_{self-collision-rejection}}{N_{total}}.$

**Table 5:** Data analysis in environment A.

Algorithm	$C_{init}$	$T_{init}$	$R_{node}$	$R_{sc}$	$T_{total}$	$C_{final}$
RRT*	3.846	3.092	15.358	2.483	24.020	3.812
Informed RRT*	3.288	2.323	15.219	2.481	16.901	3.262
MSSC-RRT*	2.411	0.057	4.466	0.010	13.066	2.408
PRM*	2.423	3.092	4.498	0.049	29.975	2.420
SAI-RRT*	2.410	0.055	4.173	0.003	13.075	2.404

**Table 6:** Data analysis in environment B.

Algorithm	$C_{init}$	$T_{init}$	$R_{node}$	$R_{sc}$	$T_{total}$	$C_{final}$
RRT*	3.869	3.608	17.721	3.306	30.781	3.844
Informed RRT*	3.815	2.753	15.587	3.135	17.719	3.554
MSSC-RRT*	2.424	0.072	15.478	0.015	13.072	2.421
PRM*	2.442	17.112	15.512	0.068	30.312	2.433
SAI-RRT*	2.417	0.068	12.816	0.013	13.091	2.412

**Table 7:** Data analysis in environment C.

Algorithm	$C_{init}$	$T_{init}$	$R_{node}$	$R_{sc}$	$T_{total}$	$C_{final}$
RRT*	5.726	11.429	18.203	3.438	40.227	5.717
Informed RRT*	5.552	10.851	15.667	3.383	17.872	5.522
MSSC-RRT*	3.324	1.835	15.599	0.019	15.011	3.299
PRM*	3.356	17.132	16.731	0.083	30.632	3.342
SAI-RRT*	3.156	1.742	12.839	0.016	14.551	3.145

**Table 8:** Average RL computation time (s) of the SAI-RRT* algorithm in different environments.

Environment	Data
sparse	2.892
narrow	2.935
dense	3.515

**Table 9:** Runtime breakdown of SAI-RRT* across different environments.

Environment	Total Planning Time	Sampling	Nearest-Neighbor Search	Collision Checking & Kinematics	DQN Refinement	Path Post-Processing
sparse	13.106	0.035	2.233	1.654	3.470	0.052
narrow	13.233	0.033	2.361	2.096	3.567	0.113
dense	14.298	0.209	2.908	2.263	4.287	0.167

**Table 10:** DQN refinement performance across different environments.

Environment	Invocation Frequency	Average Time	Worst Time	DQN Fixed Rate
sparse	1662.42	0.0021	0.0033	2.743%
narrow	1864.48	0.0019	0.0039	3.505%
dense	2013.72	0.0021	0.0044	3.780%

**Table 11:** Average minimum link clearance (m) across different environments.

Algorithm	Sparse Environment	Narrow Environment	Dense Environment
RRT*	0.0006	0.0005	0.0004
Informed RRT*	0.0007	0.0006	0.0004
MSSC-RRT*	0.0022	0.0014	0.0003
SAI-RRT*	0.0081	0.0048	0.0006

**Table 12:** Average trajectory smoothness (rad) across different environments.

Algorithm	Sparse Environment	Narrow Environment	Dense Environment
RRT*	0.246	0.416	0.559
Informed RRT*	0.239	0.480	0.564
MSSC-RRT*	0.210	0.241	0.354
SAI-RRT*	0.473	0.523	0.648

**Table 13:** Ablation results for planning efficiency and feasibility in the dense environment.

Variants	Success Rate	Planning Time	Path Length	Invalid-Node Rate
Informed RRT*	80%	18.425	5.597	19.397
Informed RRT* + Adaptive Constrained Circular Sampling	84%	11.386	3.391	17.427
Informed RRT* + Adaptive Constrained Circular Sampling + DQN Refinement	94%	14.109	3.226	13.473
SAI-RRT*	96%	14.538	3.124	12.846

**Table 14:** Ablation results for clearance (m), smoothness (rad), and collision-checking cost (s) in the dense environment.

Variants	Clearance	Smoothness	Collision-Checking Cost
Informed RRT*	0.0004	0.558	3.268
Informed RRT* + Adaptive Constrained Circular Sampling	0.0004	0.393	1.946
Informed RRT* + Adaptive Constrained Circular Sampling + DQN Refinement	0.0005	0.612	2.148
SAI-RRT*	0.0006	0.651	2.272

## Data Availability

The original contributions presented in this study are included in the article. Further inquiries can be directed to the corresponding authors.
